# Comparison of taurine, GABA, Glu, and Asp as scavengers of malondialdehyde *in vitro* and *in vivo*

**DOI:** 10.1186/1556-276X-8-190

**Published:** 2013-04-24

**Authors:** Yan Deng, Wei Wang, Pingfeng Yu, Zhijiang Xi, Lijian Xu, Xiaolong Li, Nongyue He

**Affiliations:** 1 Hunan Key Laboratory of Green Packaging and Application of Biological Nanotechnology, Hunan University of Technology, Zhuzhou, 412007, People’s Republic of China; 2 State Key Laboratory of Bioelectronics, Southeast University, Nanjing, 210096, People’s Republic of China; 3 Guangzhou The Bond Chemicals Co. Ltd., Guangzhou, 510530, People’s Republic of China

**Keywords:** Taurine, Gamma-aminobutyric acid, Glutamate, Aspartate, Scavengers, Malondialdehyde

## Abstract

The purpose of this study is to determine if amino acid neurotransmitters such as gamma-aminobutyric acid (GABA), taurine, glutamate (Glu), and aspartate (Asp) can scavenge activated carbonyl toxicants. *In vitro*, direct reaction between malondialdehyde (MDA) and amino acids was researched using different analytical methods. The results indicated that scavenging activated carbonyl function of taurine and GABA is very strong and that of Glu and Asp is very weak in pathophysiological situations. The results provided perspective into the reaction mechanism of taurine and GABA as targets of activated carbonyl such as MDA in protecting nerve terminals. *In vivo*, we studied the effect of taurine and GABA as antioxidants by detecting MDA concentration and superoxide dismutase (SOD) and glutathione peroxidase (GSH-Px) activities. It was shown that MDA concentration was decreased significantly, and the activities of SOD and GSH-Px were increased significantly in the cerebral cortex and hippocampus of acute epileptic state rats, after the administration of taurine and GABA. The results indicated that the peripherally administered taurine and GABA can scavenge free radicals and protect the tissue against activated carbonyl *in vivo* and *in vitro*.

## Background

There is a common character for all neurodegenerative diseases: all of which, such as Parkinson's disease (PD) and Alzheimer's disease (AD), are connected with neuronal apoptosis induced by oxidative stress and carbonyl stress [1,2]. Oxidative injury plays a role in the initiation and progression of epilepsy [3]. In pathophysiological situations of the brain, the high metabolic rate, low concentration of glutathione and antioxidant enzyme catalase, and high proportion of polyunsaturated fatty acids make the brain tissue and DNA particularly susceptible to oxidative and carbonyl damage causing neurodegenerative disorders [4-6]. The Maillard reaction and advanced lipid peroxidation reactions lead to the formation of advanced glycation end products (AGEs) and advanced lipoxidation end products (ALEs), whose processes have been widely documented to be responsible for the formation of various age pigment-like fluorophores and many chronic diseases, such as neuronal degenerative diseases, chronic fatigue syndrome, and physiological aging [7-11]. A variety of reactive carbonyl intermediates derived from Maillard and lipid peroxidation reactions acts as intermediates in the formation of AGEs and ALEs [12,13]. These carbonyl compounds were found to react readily with an amino group of proteins with the formation of protein aggregates, resulting in protein structural and functional alterations [14].

Malondialdehyde (MDA) is the well-studied intermediate of oxidative stress [15]. These reactive unsaturated carbonyls can target a variety of biological components, such as structural and functional proteins and nucleic acids [7,16]. MDA causes tissue injury and the depression of energy metabolism, thus representing biochemical markers for disease progression and lipid peroxidation, such as Huntington's disease [17], familial amyotrophic lateral sclerosis (ALS) [18], AD, and vascular dementia [19,20]. Recent research results suggest that schizophrenic patients exhibit increased MDA levels, which lead to neuronal damage [21]. ALEs such as MDA have been implicated in the neuronal loss observed in a variety of neuropathological cases including AD, ALS, PD, and ischemia [2,16,22]. These findings further support a role of carbonyl injury in the pathogenesis and the potential benefits of antioxidant therapy [23].

Taurine (2-aminoethanesulfonic acid) and gamma-aminobutyric acid (GABA) are both natural amino acids with wide occurrence. In the context of the neural system, taurine and GABA are inhibitory amino acid neurotransmitters, and glutamate and aspartate are excitatory amino acids. Taurine was originally described to inhibit lipid peroxidation [24]. At present, taurine has been demonstrated to protect the brain against lipid peroxidation and oxidative stress [25,26]. It has also been shown that GABA exhibits anti-hypertensive effect, activates the blood flow, and increases the oxygen supply in the brain to enhance metabolic function of brain cells [27]. Evidence suggests GABA-improved visual cortical function in senescent monkeys [28]. Decreased proportion of GABA associated with age-related degradation of neuronal function and neuronal degenerative diseases [29]. Recent study showed GABA-alleviated oxidative damage [30]. Glutamate (Glu) and aspartate (Asp) are reported to prevent cardiac toxicity by alleviating oxidative stress [31]. In this paper, it is hypothesized that several amino acids may inhibit the formation of ALEs and scavenge reactive carbonyl compounds such as MDA based on a potential carbonyl-amine reaction under physiological conditions, and its function is *in vitro* compared; also, the strong inhibition function of amino acids was investigated *in vivo*.

## Methods

### Materials and preparation

Taurine, GABA, Glu, and Asp were purchased from Sinopharm Chemical Reagent C., Ltd (Shanghai, China). 1,1,3,3-Tetramethoxypropane (TMP) and pentylenetetrazol (PTZ) were obtained from Fluka Chemie AG (Buchs, Switzerland). MDA detection kit, superoxide dismutase (SOD) detection kit, glutathione peroxidase (GSH-Px) detection kit, and total protein quantification kit (Coomassie Brilliant Blue) were purchased from Nanjing Jiancheng Bioengineering Institute (Nanjing, China). Other chemicals used were purchased from HuiHong Chemical Reagent C., Ltd. (Changsha, China).

MDA stock solution (40 mM) was prepared by hydrolyzing TMP according to a method described by Kikugawa and Beppu [32]. Thus, 0.17 mL (1.0 mmol) of TMP was added in 4 mL of 1.0 M HCl and shaken at 40°C for about 2 min. After the TMP was fully hydrolyzed, the pH was adjusted to 7.4 with 6.0 M NaOH, and the stock solution was finally made up to 25 mL with 0.2 M PBS (pH 7.4). The stock solution was checked by measuring the absorbance at 266 nm using *ϵ*_266_ = 31,500 M^−1^ cm^−1^.

### *In vitro* incubation experiments and HPLC, fluorescence, and LC/MS analysis of the incubation mixture

Several amino acids were incubated with MDA (5.0 mM) in 5 mL of 0.2 M PBS at 37°C (pH 7.4). Samples were analyzed by high-performance liquid chromatography (HPLC), fluorescence, or liquid chromatography/mass spectrometry (LC/MS) [26,33].

### Animals and drug treatment

Male or female Sprague–Dawley rats (180 to 230 g) were employed for the experiments (Shanghai Experimental Animal Center, Chinese Academy of Sciences). Five rats were kept in individual cages with water and food available *ad libitum*. The animal room was maintained at 21°C to 23°C, with a 12-h light–dark cycle. All experimental procedures were approved by the Committee of Laboratory Animals, Chinese Academy of Sciences.

Rats were intraperitoneally (i.p.) administered with 70-mg/kg dose of 1% PTZ (dissolved in saline) to induced auditory evoked potential (AEP). Control animals received the same amount of saline injections. The seizures were rated according to the following criteria [34,35]: stage 0, no response; stage I, ear and facial twitching; stage II, myoclonic jerks without upright position; stage III, myoclonic jerks, upright position with bilateral forelimb clonus; stage IV, clonic-tonic seizure; and stage V, generalized clonic-tonic seizures, loss of postural control.

Experimental rats were divided into four groups as follows: group 1, rats were treated with saline; group 2, rats were i.p. injected with a dose of 70 mg/kg PTZ to induce the onset of seizures; group 3, rats were i.p. co-administered with a dose of 70 mg/kg PTZ since i.p. injected with a dose of 500 mg/kg taurine after 30 min; and group 4, rats were i.p. co-administered with a dose of 70 mg/kg PTZ since i.p. injected with a dose of 500 mg/kg GABA after 30 min. After 1 h, the animals were killed, the brains were dissected, the cerebral cortex and hippocampus tissues were removed, and blood was withdrawn. The brain tissue was rinsed in ice-cold normal saline, added to nine times ice-cold normal saline, homogenized, and centrifuged at 5,000×*g* for 15 min at 4°C. The blood was centrifuged at 3,000×*g* for 15 min. The supernatant and serum were obtained and stored in a −20°C refrigerator for MDA assays and antioxidant enzymes' (SOD, GSH-Px) activity assays. The protein concentration was determined by Coomassie Brilliant Blue method.

### MDA assay and antioxidant enzyme activity measurement

The MDA and antioxidant enzymes' (SOD, GSH-Px) activity of the cerebral cortex and the hippocampus tissue and blood from PTZ-induced AEP were evaluated by MDA assay and antioxidant enzymes' (SOD, GSH-Px) kits according to the manufacturer's instructions.

### Statistics

Data were shown as mean ± S.E.M. Statistical evaluation was carried out by one-way analysis of variance (ANOVA) followed by Scheffe's multiple range tests. *P* < 0.05 was considered to be significant.

## Results

### Incubation products assayed by HPLC and fluorescence

**Figure 1:**
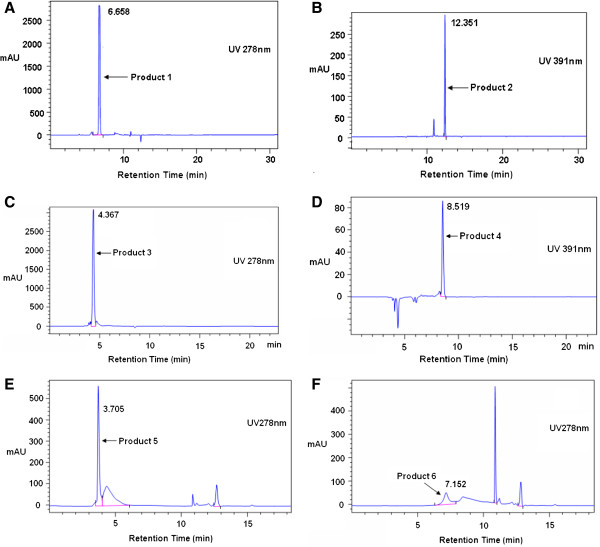
**Principal reaction products.** Taurine + MDA, GABA + MDA, Glu + MDA, and Asp + MDA separated by HPLC analysis. Taurine, GABA, Glu, and Asp (5.0 mM) were incubated with MDA (5.0 mM) in 0.2 mM PBS (pH 7.4) at 37°C for 24 h. The principal reaction products of taurine + MDA separated by HPLC analysis were observed at 278 (**A**) and 391 nm (**B**). The principal reaction products of GABA + MDA separated by HPLC analysis were observed at 278 (**C**) and 391 nm (**D**). The principal reaction products of Glu + MDA and Asp + MDA separated by HPLC analysis were observed at 278 (**E**) and 278 nm (**F**).

The mixture was separated at acidic pH through HPLC and fluorescence after amino acids (5.0 mM) were incubated with MDA (5.0 mM) in 0.2 M PBS, pH 7.4, at 37°C for 48 h. Because the standard absorbance spectrum of MDA showed a peak at 266 nm under a neutral condition and a peak at 245 nm under an acidic condition while taurine had no absorption in the range of 200 to 400 nm, products should show a peak at approximately 245 nm. By choosing the wavelengths at 274 to 278 nm, the first new products (products 1, 3, 5, and 6) were observed with the retention time of 6.658 min (Figure 1A), 4.367 min (Figure 1C), 3.705 min (Figure 1E), and 7.152 min (Figure 1F). The second new products (products 2 and 4) displayed simultaneous ultraviolet absorbance at 231 to 236 nm, 262 to 263 nm, and 391 to 394 nm with the retention time of 12.351 min (Figure 1B) and 8.519 min (Figure 1D). The first new product did not show any fluorescence, while the second new product showed a stable lipofuscin-like blue (excitation wavelength (Ex) 392 to 395 nm/emission wavelength (Em) 456 to 460 nm) fluorescence. The UV absorption maxima and fluorescence Ex/Em values of MDA, amino acids, and different products are shown in Table 1. These observations suggest that taurine or GABA reacts rapidly with MDA; in comparison, the reaction of Glu or Asp with MDA is difficult under supraphysiological conditions.

**Table 1:** UV absorption maxima and fluorescence Ex/Em values

**Compound**	**UV absorption maxima (nm)**	**Fluorescence Ex/Em (nm)**
MDA	245	No
Taurine	No	No
GABA	No	No
Glu	No	No
Asp	No	No
Product 1	278	No
Product 2	236, 263, 391	392/456
Product 3	274	No
Product 4	231, 262, 394	395/458
Product 5	276	No
Product 6	276	No

Values of the starting materials and products observed by incubation of taurine + MDA, GABA + MDA, Glu + MDA, and Asp + MDA for 48 h.

### Identification of reaction products by LC/MS

**Figure 2:**
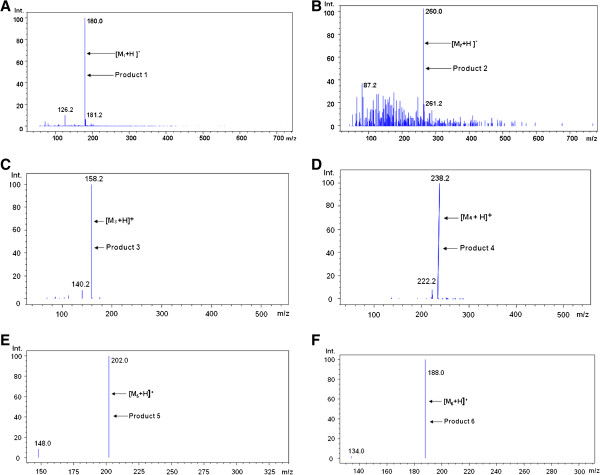
**LC/MS analysis.** Principal reaction products of taurine + MDA, GABA + MDA, Glu + MDA, and Asp + MDA after incubating for 48 h. (**A**) and (**B**) were the mass spectra of principal reaction products of taurine+MDA; (**C**) and (**D**) were those of GABA+MDA; (**E**) was that of Glu +MDA; (**F**) was that of Asp + MDA.

**Figure 3:**
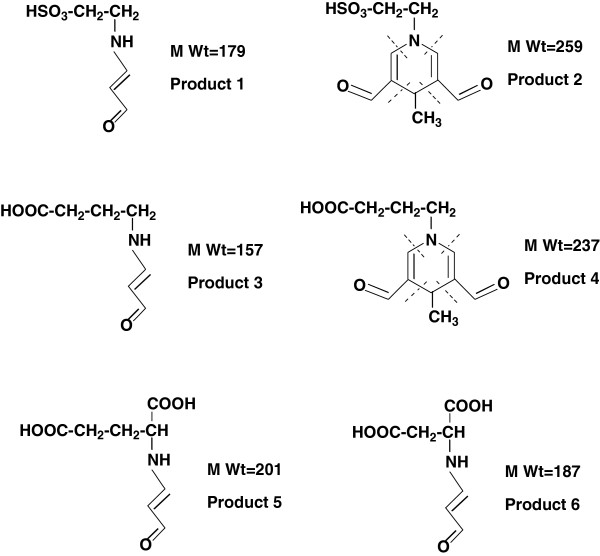
**Proposed structures.** Taurine + MDA, GABA + MDA, Glu + MDA, and Asp + MDA reaction products. Dotted lines indicate bonding positions during the product formation.

The reaction products were identified using LC/MS after the mixtures of amino acids and MDA were incubated for about 48 h. The mixture of taurine + MDA was analyzed that a total ion current chromatogram in comparison with a DAD chromatogram and the mass spectrum corresponding to the retention time of product 1 was *m*/*z* 180.0 [M_P1_ + H]^+^ (Figure 2A). Similarly, the mass spectrum corresponding to product 2 was *m*/*z* 260.0 [M_P2_ + H]^+^ (Figure 2B). After the mixture of GABA and MDA was incubated, the mass spectrum corresponding to the retention time of product 3 was *m*/*z* 158.2 [M_P3_ + H]^+^ (Figure 2C). Similarly, the mass spectrum corresponding to product 4 was *m*/*z* 238.2 [M_P4_ + H]^+^ (Figure 2D). The mixture of Glu + MDA and Asp + MDA was analyzed. The mass spectrum corresponding to the retention time of product 5 was *m*/*z* 202.0 [M_P5_ + H]^+^ (Figure 2E) and that of product 6 was *m*/*z* 187.0 [M_P6_ + H]^+^ (Figure 2F). The molecular structures of different products can be illustrated in Figure 3 according to the molecular weight and the knowledge in the related research field [7,13,31,36,37]. The formation mechanism of products 2 and 4 was similar to that of the other fluorescent dihydropyridine derivatives, which are clearly elaborated by Kikugawa and Beppu and confirmatively reviewed by Esterbauer et al.

### Comparison of the formation of reaction products of taurine, GABA, Glu, or Asp with MDA

**Figure 4:**
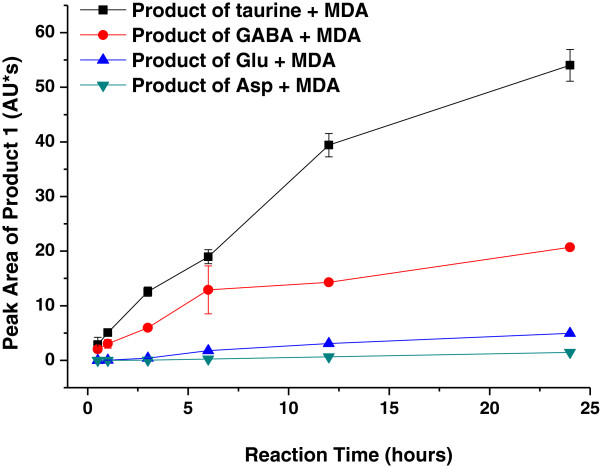
**Comparison of the formation of nonfluorescent products.** Expressed as peak area, based on the UV absorption maxima of the nonfluorescent product, during the reaction of taurine, GABA, Glu (Glu), or Asp (Asp) with MDA. Taurine, GABA, Glu (Glu), or Asp (Asp) (5.0 mM) was incubated with MDA (5.0 mM) in 0.2 mM PBS (pH 7.4) at 37°C for 24 h.

**Figure 5:**
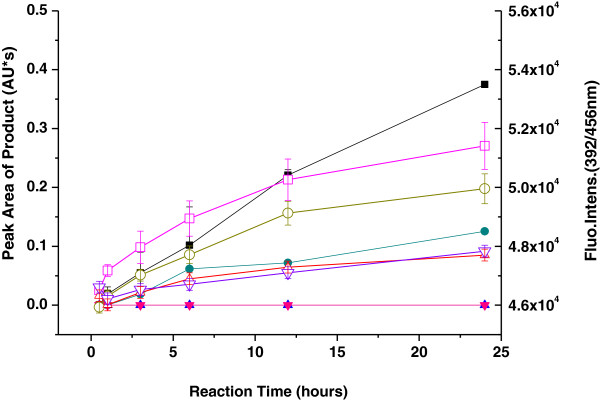
**Comparison of the formation of the fluorescent products during the reaction of taurine or GABA with MDA.** Expressed as peak area and fluorescence intensity, based on the UV absorption maxima of the fluorescent product, and fluorescence yield corresponding to the formation of the fluorescent products. Taurine or GABA (5.0 mM) was incubated with MDA (5.0 mM) in 0.2 mM PBS (pH 7.4) at 37°C for 24 h. UV absorbance of the fluorescent product of (■) taurine, (●) GABA, (▲) Glu, or (▼) Asp with MDA was measured at 391 nm. Fluorescence yield of the fluorescent product of (□) taurine, (○) GABA, (△) Glu, or (▽) Asp with MDA was measured at Ex 392 nm/Em 456 nm. Data are mean ± S.D. of triplicates.

By comparison, the fast formation of products shows that taurine can react rapidly with MDA; the reaction activity of GABA with MDA is slightly weak, but those of Glu and Asp are very slow. The relativistic mass of the nonfluorescent product after reacting between taurine and MDA is 10 times as great as that of the reaction between Glu and MDA and 40 times as great as that between Asp and MDA. Between GABA and MDA, the relativistic mass is 4 times as great as that between Glu and MDA and 14 times as great as that between Asp and MDA (Figure 4). The relativistic mass of the fluorescent products after reacting between taurine and MDA is three times than that of the reaction between GABA and MDA in 24 h (Figure 5).

### Content of MDA in PTZ-induced acute epileptic state rats

In the hippocampus of rat brains, the highest content of MDA is in AEP + normal saline (NS) group and lowest in the control + NS group. When AEP groups are treated using taurine or GABA, the MDA concentration of the taurine + AEP and GABA + AEP groups is reduced in comparison with that of the AEP + NS. The level of MDA of the AEP + NS group displays significant difference (*P* < 0.05) relative to that of the GABA + AEP group but has no statistical significance relative to that of the taurine + AEP group. MDA concentrations among the taurine + AEP, GABA + AEP, and control + NS groups have no statistical significance. In the rat cerebral cortex, the highest content of MDA is in the acute epileptic state (AEP) + NS group. MDA concentrations of the taurine + AEP, GABA + AEP, and control + NS groups are very close to each another. When AEP groups are treated using taurine or GABA, the level of MDA of the AEP + NS group has significant difference (*P* < 0.05) relative to those of the GABA + AEP and taurine + AEP groups. MDA concentrations among the taurine + AEP, GABA + AEP, and control + NS groups have no statistical significance. In the rat serums, the highest content of MDA is in the AEP + NS group. However, the MDA concentration among the taurine + AEP, GABA + AEP, and control + NS groups has no statistical significance. MDA concentrations of different groups are shown in Table 2.

**Table 2:** **Test result of MDA content of the hippocampus, cerebral cortex, and serum of every group **$\left(\bar{x}\pm s,n=7\right)$

**Group**	**Hippocampus (nmol/mg protein)**	**Cerebral cortex (nmol/mg protein)**	**Serum (nmol/mL)**
Control + NS	14.20 ± 4.54*	14.87 ± 2.64*	10.00 ± 5.19
AEP + NS	23.98 ± 4.90	25.40 ± 3.37	13.00 ± 1.92
Taurine + AEP	18.46 ± 2.27	14.55 ± 3.61*	9.55 ± 2.04
GABA + AEP	17.45 ± 1.81*	15.72 ± 7.38*	10.12 ± 2.12

Data were shown as mean ± S.E.M. Statistical evaluation was carried out by one-way analysis of variance (ANOVA) followed by Scheffe's multiple range tests: *P < 0.05, AEP + NS versus control + NS, taurine + AEP, or GABA + AEP.

### Activities of SOD and GSH-Px in PTZ-induced acute epileptic state rats

In the hippocampus of rat brains, the activity of SOD is lowest for the AEP + NS group and highest for the control + NS group. When AEP groups are treated using taurine or GABA, SOD activities of the taurine + AEP and GABA + AEP groups are heightened more than that of the AEP + NS group. SOD activity of the AEP + NS group has significant difference (*P* < 0.05) relative to that of the GABA + AEP and taurine + AEP group, but those among the taurine + AEP, GABA + AEP, and control + NS groups have no statistical significance. In the cerebral cortex of the rats, the activity of SOD is lowest in the AEP + NS group and slightly high in the control + NS group. When AEP groups are treated using taurine or GABA, the SOD activities of the taurine + AEP and GABA + AEP groups are heightened more than that of the control + NS, but those among the taurine + AEP, GABA + AEP, and control + NS groups have no statistical significance. SOD activities of different groups are shown in Table 3.

**Table 3:** **Test result of SOD activity of the hippocampus and cerebral cortex of every group **$\left(\bar{x}\pm s,n=7\right)$

**Group**	**Hippocampus (U/mg protein)**	**Cerebral cortex (U/mg protein)**
Control + NS	24.27 ± 1.83*	18.22 ± 0.31
AEP + NS	20.14 ± 0.56	16.68 ± 1.96
Taurine + AEP	23.86 ± 1.73*	22.49 ± 2.09
GABA + AEP	23.16 ± 1.38*	21.97 ± 4.93

Data were shown as mean ± S.E.M. Statistical evaluation was carried out by one-way analysis of variance (ANOVA) followed by Scheffe's multiple range tests: *P < 0.05, AEP + NS versus control + NS, taurine + AEP, or GABA + AEP.

In the hippocampus of rat brains and cerebral cortex, the activity of GSH-Px is lowest in the AEP + NS group and close to each other in the taurine + AEP, GABA + AEP, and control + NS groups. When AEP groups are treated using taurine or GABA, the GSH-Px activity of the AEP + NS group shows significant difference (*P* < 0.05) relative to those of the GABA + AEP and taurine + AEP groups, but those among the taurine + AEP, GABA + AEP, and control + NS groups have no statistical significance. GSH-Px activities of different groups are shown in Table 4.

**Table 4:** **Test result of GSH-Px activity of the hippocampus and cerebral cortex of every group **$\left(\bar{x}\pm s,n=7\right)$

**Groups**	**Hippocampus (U/mg protein)**	**Cerebral cortex (U/mg protein)**
Control + NS	26.21 ± 1.30*	32.14 ± 10.97*
AEP + NS	14.55 ± 2.07	13.90 ± 2.52
Taurine + AEP	28.17 ± 3.11*	36.68 ± 12.90*
GABA + AEP	26.12 ± 2.97*	37.65 ± 8.47*

Data were shown as mean ± S.E.M. Statistical evaluation was carried out by one-way analysis of variance (ANOVA) followed by Scheffe's multiple range tests: *P < 0.05, AEP + NS versus control + NS, taurine + AEP, or GABA + AEP.

## Discussion

Taurine is widely applied as an antioxidant or dietary supplement and is demonstrated to reduce significantly MDA levels in the serum and/or tissue [38]. GABA is widely applied as an additive [26]. Similarly, it is reported that Glu and Asp can prevent cardiac toxicity by alleviating oxidative stress [30].

Our results demonstrate that taurine or GABA reacts rapidly with MDA, and the reaction of Glu or Asp with MDA under supraphysiological conditions is difficult (Figures 1 and 2). The observations are consistent with the hypothesis that amino acids act as a sacrificial nucleophile, trapping reactive intermediates [36,37]. Scavenging carbonyl function of four amino acids is shown in Figures 4 and 5. The strong inhibition effect of taurine and GABA on MDA and the fast formation of products show that taurine and GABA can react rapidly; however, the reaction of Glu or Asp with MDA is very weak under supraphysiological conditions due to its different chemical structures (Table 1, Figure 3). In addition, if it is thought of four amino acids in the context of the neural system, taurine and GABA are important inhibitory amino acid neurotransmitters, and Glu and Asp are significant excitatory amino acid neurotransmitters. Glu and Asp uptake induce excitotoxicity, thereby causing oxidative stress and further lipid peroxidation [6]. MDA-related carbonyl stress injures neurons by triggering Ca^2+^ influx and calcium overload [39]. Indeed, it is possible that only taurine and GABA prevent neurons from damage with anticarbonylation toxic function besides inhibiting neuron superexcitation [40]. Also, studies [41] thought GABA treatment could prolong survival of transplanted β cells. MDA was considered to suppress cerebral function by breaking homeostasis between the excitation and inhibition [42]. However, MDA content in the brain tissue is enhanced dramatically to as high as 10 to 30 μm under pathophysiological conditions [43], such as aging and neurodegenerative diseases [44,45]. Thus, *in vivo* system, these results are considered if taurine and GABA can scavenge active carbonyl besides MDA in neural tissues or cells such as the epileptic focus [3] accumulated chemicals on their membrane. Here, taking AEP for example, the neuroprotective effects of taurine and GABA are investigated on peroxidation of the AEP model.

Our results have shown that MDA concentration was elevated and SOD activity decreased in the AEP rats. After administration of taurine and GABA in the cerebral cortex and hippocampus of AEP rats, the level of MDA was decreased significantly (Table 2), and the activities of SOD and GSH-Px were increased significantly. However, two administration groups had no statistical difference from each other as well as with the normal group (Tables 3 and 4). The result indicated that the peripherally administered taurine and GABA can scavenge free radicals and protect the tissue against active carbonyl harm.

## Conclusions

Our study *in vitro* demonstrates that four amino acid neurotransmitters inhibit the formation of reactive carbonyl intermediates during oxidative stress and react with MDA to form different conjugated complexes. These data illustrate taurine's or GABA's strong function to scavenge endogenous and/or further extrinsic unsaturated reactive carbonyls. In comparison, the scavenging function of Glu or Asp is very weak when reacting with MDA. The molecular mechanism of taurine's or GABA's inhibition and the investigation of its neuroprotective effects *in vivo* may prove useful for limiting the increased chemical modification of tissue proteins and cells on oxidative stress.

## Competing interests

The authors declare that they have no competing interests.

## Authors' contributions

YD and WW were responsible for carrying out the animal experimental work and the basic result analysis, as well as drafting the manuscript. PY was responsible for carrying out the HPLC analysis of the experimental work. ZX helped design the experiment and assisted with the result analysis. LX substantively edited the manuscript. XL was responsible for carrying out the incubation experiments. NH instigated and gave overall supervision to the project. All authors read and approved the final manuscript.
